# Providers’ Perspective on the Feasibility of Digital Self-Management of Blood Pressure in Refugees: Mixed Methods Study

**DOI:** 10.2196/66176

**Published:** 2025-10-24

**Authors:** Carol Gonzalez, Marcos Real, Nargis Ahmadi, Raghad Aljenabi, Lana Bridi, Nour Makarem, Job Godino, Tala Al-Rousan

**Affiliations:** 1 Herbert Wertheim School of Public Health, University of California San Diego, 9500 Gilman Drive, La Jolla, CA, 92093, United States, 1 8585342230; 2 School of Medicine, University of California San Diego, La Jolla, CA, United States; 3 University of California San Diego, La Jolla, CA, United States; 4 Mailman School of Public Health, Columbia University Irving Medical Center, New York, NY, United States; 5 Family Health Centers of San Diego, San Diego, CA, United States

**Keywords:** hypertension, refugee health, self-management, digital health, digital self-management, feasibility, blood pressure, refugee, mixed methods, public health, telemonitoring, telehealth, physician, medical assistants, real-time tracking, telemonitoring intervention, hypertension disparities

## Abstract

**Background:**

Mass displacement is a grand public health challenge. Refugees and immigrants experience a disparate hypertension burden and disparities in self-management. Successful hypertension self-management is key for improving outcomes, but research on its feasibility in refugee and immigrant health care settings is limited.

**Objective:**

This study aimed to identify clinic staff–perceived barriers to and facilitators of implementing a digital intervention for hypertension self-management among refugee and immigrant patients and to identify its feasibility and usability.

**Methods:**

Primary care physicians and medical assistants who care for refugees and immigrants in San Diego were interviewed using human-centered semistructured methods (n=18). Interviews were analyzed using an inductive approach. Usability testing for the software (Med Pro Care) was conducted with participants (n=15) to test the feasibility of real-time tracking of blood pressure (BP) home readings for hypertension self-management. Clinical staff rated their satisfaction on the System Usability Scale and the NASA Task Load Index, which measured mental workload.

**Results:**

For refugee and immigrant patients self-managing hypertension, clinical staff identified barriers and facilitators in the following areas: (1) social determinants of health increase hypertension burden among refugee and immigrant patients, (2) clinical staff face challenges to effective hypertension care for refugee and immigrant patients, (3) perceived benefits of potential intervention for self-management, and (4) perceived barriers to potential intervention for self-management. Primary care physicians completed 90% of the tasks, and medical assistants completed 83% of the tasks successfully. Most clinic staff found the software system for monitoring BP to be easy to use with an average score for usability of 4.1 of 5.0 (SD 0.4).

**Conclusions:**

Addressing identified barriers to and facilitators of self-management of hypertension is crucial to designing effective interventions in real-world refugee and immigrant health care settings. Telemonitoring interventions using software that transfers BP readings to clinical staff in real time may be feasible from the perspective of clinic staff and can address hypertension disparities in marginalized populations, such as immigrants and refugees. Addressing identified barriers to and facilitators of self-management is crucial to designing effective interventions in real-world refugee and immigrant health care settings. Our findings suggest that clinic staff view digital telemonitoring as both feasible and supportive of patient empowerment, health literacy, and improved communication—factors essential to addressing hypertension disparities in marginalized populations.

## Introduction

### Background

Hypertension holds global significance as one of the most important modifiable risk factors for cardiovascular disease. Almost one-third of the global population is diagnosed with hypertension [1], which causes 7.5 million deaths annually, and its increasing prevalence has led to greater morbidity for the global population from its sequelae, such as heart disease [2,3]. Notably, forcibly displaced populations, whose numbers are at all-time highs, including immigrants and refugees, experience a heightened burden of hypertension [4,5]. The challenges of fleeing conflict, interrupted health care access, adapting to new environments, and enduring persecution and trauma amplify the stress experienced by refugees and contribute to a higher hypertension risk [6-8]. In addition, in host societies, immigrants and refugees confront multifaceted barriers to engaging in health care, encompassing language and cultural barriers, economic barriers, and acculturation difficulties [9]. These factors contribute to this minoritized group’s poor control of hypertension [10-14]. This is reflected in empirical evidence consistently pointing to elevated hypertension rates and almost double the cardiovascular disease risk in refugees compared to other immigrants or the hosting population, irrespective of their country of origin, compared to host country citizens [5,8].

Self-management of hypertension, including both self-monitoring of blood pressure (BP) and self-titration of medication, is an evidence-based, cost-effective method for improving BP control [15,16]. Patients who self-monitor are more likely to lower their BP [17,18], have fewer BP-related office visits [19,20], improve communication with their providers regarding lifestyle factors [21], and incur significant health care cost savings compared to usual care groups [22], regardless of the number of other comorbidities [23]. In addition to the reported accuracy of self-monitoring, many providers share positive perspectives on self-titration, emphasizing its potential benefits, including improved patient-provider relationships and enhanced self-efficacy [24-26]. Given the ongoing self-management demanded by chronic conditions such as hypertension, self-titration could provide crucial support for clinics in efficiently caring for their patients and decreasing clinic visits [7,21,27].

### Objective

Providers are often responsible for health education, telemonitoring, and regular follow-up with patients for hypertension self-management, but acceptability and efficacy of digital health technologies, integration with existing electronic health records, usability, and technology support have emerged as key concerns [15,17,24,28]. Our study examined the feasibility of using technology for BP monitoring when caring for refugee and immigrant patients, and the barriers to and facilitators of this model of care. We also assessed the usability of a specific software (Med Pro Care; Withings) attached to connected BP monitors (Withings) that can help clinic staff implement telemonitoring in their clinics while receiving real-time BP readings from their patients at home [29].

## Methods

### Design

This study was part of a larger investigation into refugee and immigrant patients’ barriers to hypertension care and opportunities to improve self-management. Patient participants in that study were provided a free and validated Withings BP monitor, a Food and Drug Administration–approved, cellular-connected home BP monitor [29], and were instructed to maintain a BP diary. The data from the BP monitor were transmitted to a web-based dashboard called Med Pro Care [29]. Our part of the study examined the clinic staff’s perspective and experience with the Med Pro Care software using the data recorded from the patients in the larger study. Clinic staff participants in our study were previously trained by our staff on the use of the Med Pro Care dashboard, where they could view the charts for the patients in the larger study and where to find their BP readings in the software. The study was designed to determine the feasibility and usability of the Med Pro Care software for future research on longitudinal telemonitoring.

Our study used a human-centered design, mixed methods approach to gathering data concerning the perspectives of clinic staff on self-management of hypertension for refugee and immigrant patients and the usability of Med Pro Care for use in the clinic to help clinical staff telemonitor patients’ home BP readings. We administered semistructured interviews, a demonstration of the Med Pro Care software for reviewing patient charts, and a usability test of Withing’s Med Pro Care software, all to both medical assistants (MAs) and primary care providers (PCPs). Qualitative interviews aimed to identify clinic staff’s experiences with treating refugee and immigrant patients with hypertension to better understand the social aspect of their health care (Multimedia Appendix 1), whereas the quantitative data collected from the usability test demonstrates the effectiveness of a possible solution addressing identified social factors (Multimedia Appendices 2-4). This study was conducted at federally qualified health centers, university clinics, and private clinics across southern San Diego County, California, where most refugees and immigrants in the region have resettled [30].

### Participants

Participants included the following clinic staff: (1) MAs and (2) PCPs in San Diego County who regularly provided care for refugee and immigrant patients. We chose to ask both the same questions and combine their responses for data analysis because of growing evidence showing that multilevel interventions that involved integrated multidisciplinary care and promotion of self-management behaviors, which is done by diverse clinic staff, including providers, nurses, pharmacists, and MAs, were associated with the largest BP reductions [31,32]. Clinical staff were included if they self-reported regularly seeing refugee or immigrant patients in the San Diego region. Individuals were excluded if they did not care for patients with hypertension or self-reported not regularly caring for refugee or immigrant patients. While some participants self-identified as immigrants themselves, our study did not include a formal inquiry about previous or current citizenship or legal status.

Recruitment took place between August 2021 and November 2022 through 4 sites: (1) the Family Health Centers of San Diego (FHCSD), a federally qualified health center system that provides care for a large portion of refugees in San Diego County; (2) University of California San Diego Health System; and (3) 2 private practices in San Diego County. Study staff received contact information for potential participants through hospital directories and contacted clinic staff via email or phone. MAs were recruited via mass email to all MAs at the federally qualified health center, to which 13 MAs responded, and 2 were excluded for not working with patients with hypertension. Eleven MAs consented and participated, with 1 MA not completing the usability assessment. PCPs were referred by senior study staff who were aware of their practice. All 7 PCPs referred consented and participated, with 2 not completing the usability assessment.

### Data Collection

Study staff created standardized operating procedures to ensure all interviews were conducted the same, regardless of the study staff who conducted the interview. In this process, the study team reviewed available literature about barriers to the implementation of self-management of hypertension for refugee and immigrant patients and created questions that could illuminate the clinic staff’s perspective. The principal investigator then reviewed and approved these questions (Multimedia Appendix 1). The study coordinator (CG) then trained incoming study staff by reviewing the procedures and having new study staff shadow an interview.

Interviews were conducted in the participant’s clinic (n=15), except for 1 PCP who participated in an office belonging to research staff and 2 PCPs who participated in the qualitative interview portion of the study over Zoom and did not complete the usability testing. Interviews typically lasted about 60 minutes in total and were audio recorded and later transcribed for qualitative analysis. Demographic information was collected from participants. Interview questions were the same for both MAs and PCPs and asked about their understanding of barriers to and facilitators of health care for refugee and immigrant patients, patient–clinic staff relationships, and the clinic staff’s opinions of self-management of hypertension, including both self-monitoring and self-titration of antihypertensive medication. Although self-titration was not tested in the larger study, thus data were not available for our usability tests, participants were asked about their opinions on whether this was something that could help their refugee and immigrant patients because it has been previously shown to help self-management efficacy, such as in the TASMIN (Targets and Self-Management for the Control of Blood Pressure) trial where self-monitoring BP and an individualized self-titration algorithm improved systolic BP for patients in the United Kingdom [15,16]. Interview questions were the same for both MAs and PCPs because of their similar exposure to the social factors that were examined by the questions. While the focus was on refugee patients, question wording was modified to include immigrants for clinic staff who expressed more experience with immigrant patients rather than specifically refugees. Study staff then demonstrated the Med Pro Care software, which included administrative and monitoring tasks. Participants were then asked to complete a usability test of the software, where MAs were tested on more administrative tasks, such as registering patients and leaving notes on accounts, and PCPs were tested on more in-depth review of BP readings. Both MAs and PCPs were asked to complete 5 tasks. The usability test was screen and audio recorded using the TURF 4.0 software (McWilliams School of Biomedical Informatics) [33]. Participants then completed the System Usability Scale [34] and the NASA Task Load Index [35] for self-reported satisfaction and usability. Study staff then asked participants if they had any additional feedback on how the software could be used in their own clinics.

### Data Analysis

Qualitative and quantitative data were analyzed separately. The semistructured interviews were analyzed using an inductive thematic approach to capture participants’ experiences as comprehensively and accurately as possible [36]. To improve the credibility of the analysis, investigator triangulation was implemented as follows [37]: (1) 2 study staff (CG and MR) independently coded a set of transcripts (n=3), (2) then had multiple meetings to define a codebook and established intercoder agreements through the subjective assessment method [36]. The codebook was then reviewed by the principal investigator, finalized, and distributed to other researchers (NA and RA) who coded the remaining transcripts. Two study staff (CG and LB) analyzed the codes for recurrent themes following Crabtree and Miller’s 5-step interpretive process [38]. Qualitative data were analyzed using Atlas. Using Microsoft Excel, 1 research team member (CG) performed basic descriptive statistical analysis on the demographic data, task completion, total clicks (collected from TURF software), and satisfaction questionnaires. For task completion, participants were given 1 point for successfully completing a task and half a point for partially completing a task. A final score was given as a percentage of tasks completed. Averages were then taken of all MAs scores and all PCPs scores separately. For the items that were negatively worded in the System Usability Scale, responses were reverse coded so that all items were aligned in the same direction, with higher scores being more favorable.

### Ethical Considerations

The University of California San Diego Human Research Protection Program (#200063) granted institutional review board approval. All participants consented before participation and were compensated with a US $35 Visa gift card for their time. All participant data were fully deidentified prior to analysis to protect individual privacy and confidentiality. As part of our study, participants examined BP measurements from a larger investigation into refugee and immigrant patients’ hypertension self-management (IRB#200063).

## Results

### Participant Characteristics

Data on participant demographics are listed in Table 1. MAs made up 61.1% (n= 11) of the 18 participants. Most participants were female (n=15, 83.3%). The 3 male participants were all PCPs. One PCP was a nurse practitioner, whereas all others were physicians. All MAs were associated with FHCSD, whereas 42.9% (3/7) of PCPs were associated with FHCSD. The average age of participants was 38.9 (SD 11.9) years. Most participants self-identified as White (n=6, 33.3%), Hispanic (n=4, 22.2%), or Middle Eastern/North African (n=3, 16.7%). Besides English, the most commonly spoken languages were Spanish (n=9, 50%) and Arabic (n=6, 33.3%).

**Table 1.** Demographics of participants (N=18).

Demographics	Values, n (%)
Position	
PCP^a^	7 (38.9)
MA^b^^,^^c^	11 (61.1)
Clinic	
FHCSD^d^	14 (77.8)
Other clinics	4 (22.2)
Gender	
Male	3 (16.7)
Female	15 (83.3)
Race/ethnicity	
White (non-Hispanic)	6 (33.3)
Black (non-Hispanic)	2 (11.1)
Hispanic	4 (22.2)
Other	6 (33.3)
Middle Eastern/North African	3 (16.7)
Indian American	1 (5.6)
Multiracial	2 (11.1)
Languages spoken	
English only	4 (22.2)
Spanish	9 (50.0)
Arabic	6 (33.3)
French	2 (11.1)
Aramaic	1 (5.6)
Chaldean	1 (5.6)
German	1 (5.6)
Russian	1 (5.6)
Turkish	1 (5.6)
Level of education	
High school graduate or equivalent	1 (5.6)
Some college, no degree	6 (33.3)
Associate’s degree	2 (11.1)
Bachelor’s degree	2 (1.1)
Master’s degree^e^	1 (5.6)
Medical Doctor	5 (27.8)
Doctor of Osteopathic Medicine	1 (5.6)

a PCP: primary care physicians.

b All MAs were associated with FHCSD.

c MA: medical assistant.

d FHCSD: Family Health Centers of San Diego.

e Nurse practitioner.

### Barriers and Facilitators Identified From Interviews

The interviews from this study, both MAs and PCPs, yielded 4 themes for barriers and facilitators: (1) social determinants of health increase hypertension burden among refugee and immigrant patients, (2) clinical staff face challenges to effective hypertension care for refugee and immigrant patients, (3) perceived benefits of potential intervention for self-management, and (4) perceived barriers of potential intervention for self-management.

### Social Determinants of Health Influence Hypertension Burden Among Refugee and Immigrant Patients

All participants, including both MAs and PCPs, described hypertension as a burden among many of their refugee patients and described various factors unique to these patients influencing its management. Some described how patients’ attitudes toward the disease limit care, including how patients may lack self-efficacy or motivation to make necessary changes to their care regimen. For example, when asked if refugee patients often followed the advice or directions given by the physician, an MA stated:

*some [patients] are stuck in their ways. And they don’t feel like changing is gonna help them either way, so they just continue to do whatever they’re doing*.

[MA]

Participants also acknowledged how the experience of forced displacement along with its associated stress and trauma can exacerbate hypertension:

*Especially in a refugee population, we do see stress causing high blood pressure pretty directly. And so, to talk about any traumas they may have experienced, and have a way to treat those, [is important]... just really create an open conversation about that because I think it’s not often talked about*.

[PCP]

They described the health care barriers refugee and immigrant patients face such as language and cultural barriers:

*...oftentimes they come untreated. I feel like it’s similar to other patient populations, but the difference is that the language barrier and education has hindered treatment in the past, or they stop treatment for periods of time*.

[PCP]

Participants also noted that poor health literacy and low socioeconomic status can contribute to difficulty with managing hypertension:

*Patients don’t have the supplies to take a proper blood pressure or they don’t have the correct information [to self-manage hypertension]*.

[PCP]

### Clinical Staff Face Challenges to Effective Hypertension Care for Refugee and Immigrant Patients

Common barriers faced by clinical staff when treating refugees and immigrants included effective communication, health literacy, adherence, and monitoring. Participants noted physician-patient communication was a critical variable in delivering effective care. For example, when a PCP was asked to mention a specific worry regarding their patients and the care they receive, they noted:

*Yeah, the language barrier, them not understanding the education part of it, taking medication incorrectly*...

[PCP]

Furthermore, both MAs and PCPs expressed concern that not all their refugee and immigrant patients completely understand the instructions that are given, or clinical staff do not have enough time to explain everything. This leads to miscommunication and confusion for patients, which can lead to uncontrolled and untreated hypertension, worsening patient health. Even with assistance from interpreters, participants highlighted that patients would often struggle to understand the concept and how to conduct BP checks. This miscommunication was emphasized when an MA stated that:

*...even with the interpreters they do struggle to check the BP. I know some people receive the machine but they don't know how to use the machine and they don't let us know they don’t know how to use it so there is always miscommunication between patient and provider or medical staff.*

[MA]

Clinical staff were also concerned that their refugee and immigrant patients are nonadherent to treatment for a multitude of reasons, including access to monitoring technology, ability and willingness to take medication, and willingness to make lifestyle changes. Indeed, participants discussed how some patients would veer off the established plan and take medications in a manner that was not agreed on with the physician. When discussing the burden of hypertension within refugee patients, a PCP mentioned:

*I actually have a lot of patients that they just go off the plan on their own because they think like they know it and they stop medications randomly. They take extra from others without like running it by me and they don’t even admit it until I try to get it out of an individual.*

[PCP]

Another significant variable that may exacerbate the prevalence of hypertension among refugee patients was concerns over patient adherence to treatment. Many refugee patients had fears regarding the consequences of undergoing medical intervention from clinic staff and thus were noted as being less compliant. This was highlighted when an MA was asked about concerns over medical treatments toward refugees and stated that:

*...not wanting to take the medication because they are scared, or I know some patients get scared of taking medications and think it might affect the organs.*

[MA]

### Perceived Benefits of Potential Intervention for Self-Management

When asked about participation in a hypertension self-management intervention, participants largely endorsed potential benefits for their patients, with 15 participants expressing interest in this type of intervention. Interviewed participants often expressed the desire to not only educate their patients but also encourage active participation in treatment planning and self-management of their condition. To facilitate patient adherence and hypertension self-management, participants would often try to educate refugee and immigrant patients on the significance of checking BP consistently at home and the concept of following trends. Physicians would also explain how hypertension does not always present with noticeable symptoms and can eventually lead to severe consequences if not managed on time. When discussing methods that help patients adhere to their medications, a physician stated the following:

*Explaining to them the rationale behind checking the blood pressure at home and the trend and also educating the patient about hypertension being a silent killer. And that most people with high blood pressure have no symptoms initially.*

[PCP]

With regard to the Med Pro Care device, most of the participants interviewed accepted self-management for hypertension to include both self-monitoring and self-titration. They believed that it more actively involves and empowers patients to be more in control of their health. In addition, self-management would reduce clinic burden, as patients would not have to come to the clinic as much because they would be monitored more consistently. For example, after trying out the Med Pro Care device, a physician was asked whether the device would be beneficial in the clinic and the physician responded with:

*I think, overall, that is a really beneficial way to empower patients, and to not have to have them come back to the clinic as frequently when access is already pretty hard. And then, just to feel like they are in more control of their medications.*

[PCP]

As both self-monitoring and self-titration have been successful for the management of other diseases, such as diabetes, most participants believed that with standardized protocols, it can also be successful for self-management of hypertension. Furthermore, self-management through Med Pro Care was overall noted as being a safe method to monitor and manage hypertension with the possibility of a reduction in adverse outcomes in the long term. This sentiment was explicitly noted by a physician when questioned about the potential benefits of the implementation of the Med Pro Care device:

*I think that'll make for quicker, I guess, management of their high blood pressure. Minimize, you know, the adverse effects of like, you know, strokes, kidney damage, other things like that... I think it’s very safe, this method of blood pressure monitoring by the patient themselves. It’s safe and natural, and I think if we can implement more of that make that more available overall it’s going to improve patient care and lessen adverse outcomes.*

[PCP]

Despite acknowledging factors that lead to poor BP control, participants noted how family support and caregivers of refugee and immigrant patients play a significant role in their care. For example, families often attend appointments with patients and assist with BP monitoring, facilitating self-management and improved BP control:

*I think immigrant patients tend to have a lot of family support and their kids often just help them and so they can pretty much take care of like downloading the app and checking their BP every day.*

[MA]

### Perceived Barriers to Potential Intervention for Self-Management

Two participants expressed concerns about implementing both self-monitoring and self-titration, mainly noting lack of success in patients self-titrating their hypertension medication. In addition, there was some concern over a possible increased workload because of monitoring an increased number of available readings. One participant was specifically concerned about legal liability if the clinic missed concerning BP readings and the patient did not reach out to the clinic or go to the hospital:

*My concern is that... Let’s say you did not see that alert. I mean, I have patients every day. I'm going to be honest. I don't know if I have time to check this every day... I don’t want extra liability.*

[PCP]

There was also a concern that patients would not be motivated enough to commit to their treatment plans without coming into the doctor’s office regularly and would not remain adherent to medication and monitoring enough to maintain control over their BP. Although these concerns were raised, most participants remained optimistic that self-management of hypertension was feasible with protocols and training.

### Results of Task Study

MAs were able to complete approximately 69% of the 5 tasks given to them, which included more administrative tasks such as how to register patients in the system and filtering through patients. Technical errors occurred with patient registration during many of the usability tests, resulting in all but 2 MAs being unable to complete the registration task, which could have influenced MA ratings of usability. Excluding this task, MAs were able to complete approximately 83% of tasks, with the next lowest score being in finding specific BP readings. PCPs completed approximately 90% of the 5 tasks, which were more focused on monitoring patients’ readings over time. MAs completed all tasks with an average of 90.75 (SD 41.82) clicks, and PCPs completed all tasks with an average of 129.20 (SD 85.86) clicks. The average time to complete tasks for MAs was 6 minutes and 5 seconds (SD 2 min and 28 s), whereas the average time for PCPs was 4 minutes and 35 seconds (SD 48 s). With an average usability score of 4.1 of 5.0 (SD 0.4), participants believed the system was usable, as presented in Table 2, with the highest ratings in being able to quickly learn the software and it being easy to use. Results from the Task Load Index (Table 3) also indicate that participants believed that the tasks were not burdensome or stressful. MAs and PCPs had similar ratings of usability and satisfaction, apart from 1 MA who rated the physical demand and how hard they had to work as higher than others.

**Table 2.** System Usability Scale results.

System Usability Scale^a^	MA^b^, mean (SD)	PCP^c^, mean (SD)	All participants, mean (SD)
I think that I would like to use Med Pro Care frequently.	4.4 (1.2)	4.0 (1.0)	4.3 (1.0)
I found Med Pro Care unnecessarily complex.^d^	4.2 (1.4)	3.0 (0.3)	3.8 (0.9)
I thought Med Pro Care was easy to use.	4.4 (1.4)	4.4 (1.2)	4.4 (1.3)
I think that I would need the support of a technical person to be able to use Med Pro Care.^d^	4.3 (1.2)	3.6 (0.8)	4.1 (1.0)
I found the various functions in Med Pro Care were well integrated.	4.3 (1.1)	4.2 (1.4)	4.3 (1.1)
I thought there was too much inconsistency in Med Pro Care.^d^	4.2 (1.0)	4.2 (1.4)	4.2 (1.1)
I would imagine that most people would learn to use Med Pro Care very quickly.	4.6 (1.3)	4.4 (1.2)	4.5 (1.2)
I found Med Pro Care very cumbersome to use.^d^	3.1 (0.4)	4.0 (0.9)	3.4 (0.5)
I felt very confident using Med Pro Care.	4.4 (1.2)	3.4 (1.4)	4.1 (1.2)
I needed to learn a lot of things before I could get going with Med Pro Care.^d^	4.0 (1.0)	4.2 (1.3)	4.1 (1.0)

a All questions on a scale from 1 to 5, 1=strongly disagree, 2=disagree, 3=neither disagree nor agree, 4=agree, and 5=strongly agree.

b MA: medical assistant.

c PCP: primary care provider.

d Negatively worded items were reverse coded so that higher scores indicate more positive responses.

**Table 3.** NASA Task Load Index.

NASA Task Load Index^a^	MA^b^, mean (SD)	PCP^c^, mean (SD)	All participants, mean (SD)
How mentally demanding were the tasks?	0.0 (0.0)	6.8 (7.8)	5.4 (7.4)
How physically demanding were the tasks?	9.5 (13.4)	3.5 (4.7)	5.5 (7.7)
How hurried or rushed was the pace of the tasks?	2.7 (2.5)	5.3 (6.7)	4.1 (5.1)
How successful were you in accomplishing what you were	6.2 (7.4)	5.3 (8.5)	5.8 (7.4)
How hard did you have to work to accomplish your level of performance?	9.0 (12.7)	4.8 (6.1)	6.0 (7.5)
How insecure, discouraged, irritated, stressed, and annoyed were you?	0.7 (0.6)	1.3 (2.5)	1.0 (1.8)

a All items were measured on a scale from 0 to 20, with 0 being the least amount of demand or effort and 20 being the greatest amount of demand or effort, except the performance item where perfect is 0 and failure is 20.

b MA: medical assistant.

c PCP: primary care provider.

On the basis of the follow-up questions following the usability testing, participants were generally satisfied with the software, especially in areas such as ease of use and being able to learn the system easily. Participants expressed concern over logistical issues such as how well this would integrate with currently existing electronic health records, who was responsible for keeping up with the increased number of measurements, and legal liability.

## Discussion

### Principal Findings

There was a general understanding by local clinic staff that refugees and immigrants experience pre- and postmigration stressors that negatively impact health, including increasing the risk of uncontrolled hypertension, and complicating treatment. Our study showed that addressing these unique barriers is essential for effective hypertension management. The Med Pro Care software was generally accepted by our sample and can be used in the clinic to facilitate self-monitoring of BP. Digital self-monitoring can enhance patient-clinic communication and support chronic disease self-management. Our study adds to existing literature by examining the clinic staff’s perspective in treating refugee and immigrant patients and what can be done in the clinic to better address their specific needs.

Participants emphasized cultural and linguistic barriers, access, and patient empowerment as key priorities. Consistent with prior research, an approach that is sensitive to the cultural context and acknowledges the unique experiences of refugee and immigrant patients is pivotal in establishing effective communication and fostering patient engagement [39]. For refugees and immigrants, who must navigate unfamiliar health care systems in new countries, strategies that improve communication and enhance patient self-efficacy are crucial to long-term chronic disease self-management [40].

Clinic staff generally believed that self-management of hypertension can help empower patients by improving communication, self-efficacy, and health literacy, thereby potentially enhancing hypertension management. This aligns with other studies, which show self-management enhances health awareness and literacy, although few have focused on refugee and immigrant populations [41,42]. Our results, therefore, provide a unique perspective because the clinic staff interviewed have first-hand accounts of the barriers and facilitators toward health management in refugee and immigrant populations. Further research should directly examine the perspective of refugee and immigrant patients to inform clinical implementation.

Self-management has shown great potential in controlling hypertension; however, scarce resources and logistical concerns have slowed its adoption into many clinics [26]. Clinic staff noted that relying on self-management could reduce clinic burden by reducing the need for patients to come into the clinic as frequently. The usability of the Withing’s Med Pro Care software was overall well received and considered easy to use and requiring minimal training, which could support efficient integration into clinical practice.

Perceived challenges of self-management included the clinic’s ability to handle an increased load of information and safety concerns over self-titration of medications. Participants noted that Med Pro Care’s ability to flag concerning readings and display trends could address some of these concerns. Liability concerns may be mitigated by patient education and standardized protocols. In addition, clinical trials have shown self-titration to be feasible and safe for patients with hypertension [28,40,43]. Med Pro Care can facilitate communication between patients and clinics through regular BP readings and can increase the safety of self-titration of antihypertensive medications.

Our study recruited participants from 3 different types of clinics: a federally qualified health center, a university clinic, and 2 private practices. Despite differences in electronic medical records and resources, clinic staff across settings shared similar perspectives, showing shared experience and suggesting broad applicability of Med Pro Care.

Recent studies from China have supported multimodel digital interventions for the management of hypertension [44,45]. A similar intervention in Pakistan based on the Health Belief Model included reminders, education, and 24/7 individual support, which improved medication adherence and systolic BP [46]. These studies share similarities in addressing social factors by addressing individual needs for care and support but lack a focus on the unique needs of refugee and immigrant patients. Our study highlights this gap and supports future testing of digital hypertension self-management platforms tailored to refugee and immigrant populations.

### Limitations

Despite being among the few in the field who researched refugee and immigrant population providers, our study had notable limitations. First, our sample was limited to 1 county and may not reflect clinics elsewhere. Second, clinic staff with heavier patient loads may have been underrepresented due to the lengthy interview process. Nonetheless, San Diego’s large refugee and immigrant population provides valuable insights, and future studies should evaluate digital self-management interventions in broader settings to avoid exacerbating disparities.

### Conclusions

While self-management interventions have been shown to be effective in managing hypertension, it is essential to incorporate input from clinic staff that care for refugee and immigrant populations to understand the real-world implications of eHealth interventions. Clinic staff generally found the Med Pro Care software easy to use and feasible for integration into care, supporting the role of digital tools in addressing hypertension management among refugees and immigrants. Our study demonstrated that the refugee and immigrant communities have unique needs in hypertension management related to access, language, and clinic support. The implementation of self-management is supported by clinic staff because of its potential to empower patients by facilitating autonomy and a more active role in patient engagement in health care. Hypertension self-management interventions should focus on increasing health literacy, self-efficacy, improving access to health care, and leveraging digital health to streamline workflows for clinics and enhance communication with patients. Future studies should focus on the development and evaluation of contextually tailored hypertension self-management interventions that are responsive to the unique needs of the heterogeneous US refugee and immigrant population.

## Supplementary material

10.2196/66176 Multimedia Appendix 1 Qualitative interview.

10.2196/66176 Multimedia Appendix 2 Tasks completed by participants.

10.2196/66176 Multimedia Appendix 3 System Usability Survey.

10.2196/66176 Multimedia Appendix 4 NASA Task Load Index.

## Abbreviations

BP: blood pressure
FHCSD: Family Health Centers of San Diego
MA: medical assistant
PCP: primary care provider
TASMIN: Targets and Self-Management for the Control of Blood Pressure
