# Supplementary figures and images for: Providers’ Perspective on the Feasibility of Digital Self-Management of Blood Pressure in Refugees: Mixed Methods Study
Source: JMIR Hum Factors. 2025 Oct 24;12:e66176. doi: 10.2196/66176 (PMC12551932; doi:10.2196/66176)

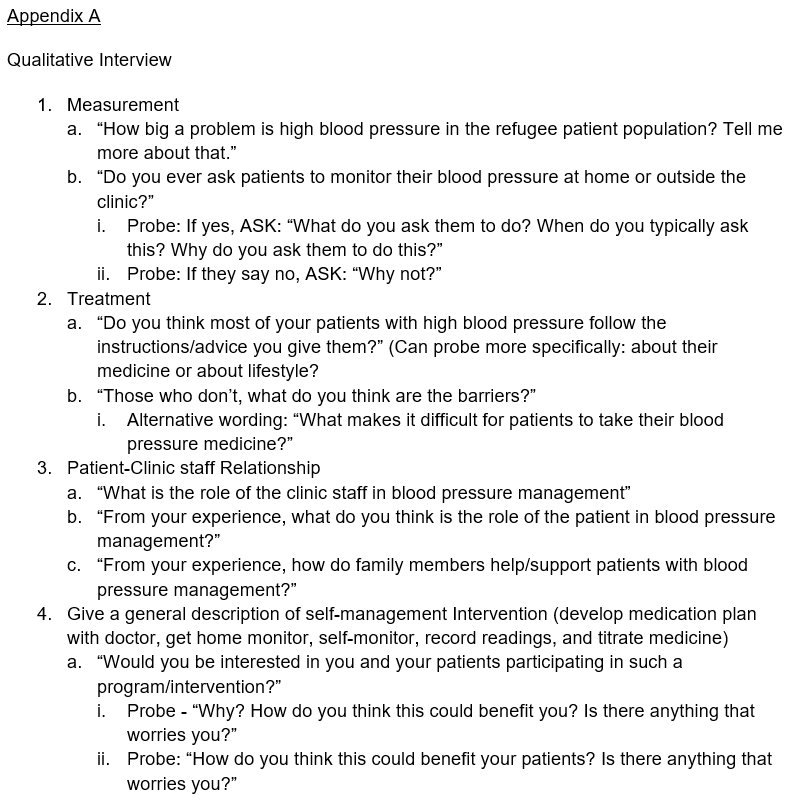

Supplement: Multimedia Appendix 1 [file humanfactors-v12-e66176-s001.png]

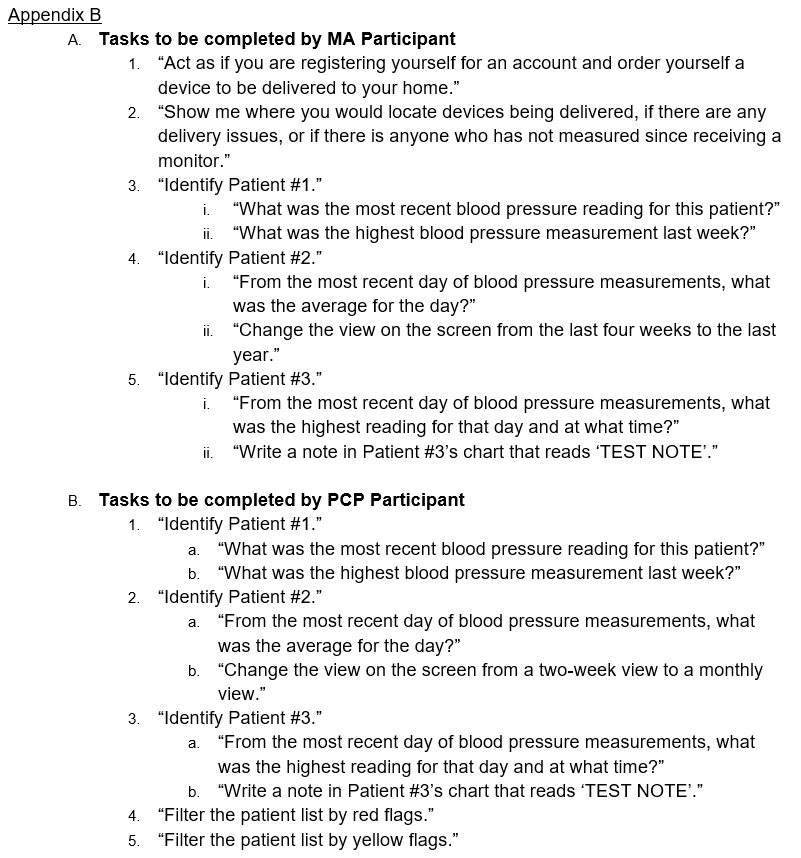

Supplement: Multimedia Appendix 2 [file humanfactors-v12-e66176-s002.png]

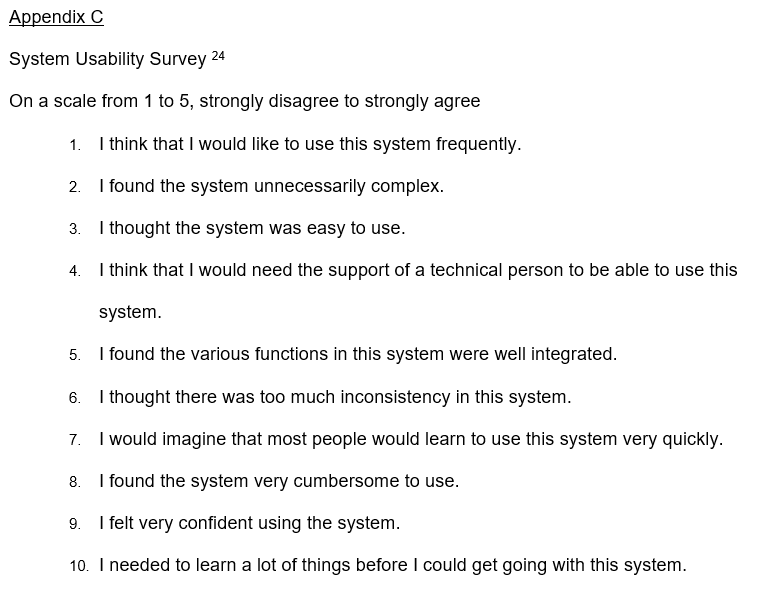

Supplement: Multimedia Appendix 3 [file humanfactors-v12-e66176-s003.png]

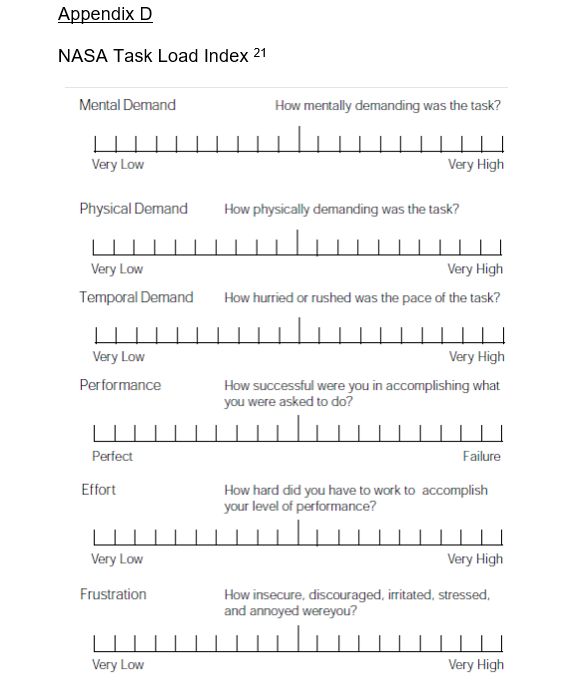

Supplement: Multimedia Appendix 4 [file humanfactors-v12-e66176-s004.png]
